# Contraceptive use among adolescent and young women in North and South Kivu, Democratic Republic of the Congo: A cross-sectional population-based survey

**DOI:** 10.1371/journal.pmed.1003086

**Published:** 2020-03-31

**Authors:** Sara E. Casey, Meghan C. Gallagher, Jessica Kakesa, Anushka Kalyanpur, Jean-Baptiste Muselemu, Raoza Vololona Rafanoharana, Nathaly Spilotros

**Affiliations:** 1 RAISE Initiative, Heilbrunn Department of Population and Family Health, Mailman School of Public Health, Columbia University, New York, New York, United States of America; 2 Save the Children US, Washington, DC, United States of America; 3 International Rescue Committee, Kinshasa, Democratic Republic of the Congo; 4 CARE USA, Atlanta, Georgia, United States of America; 5 Save the Children, Goma, Democratic Republic of the Congo; 6 CARE, Goma, Democratic Republic of the Congo; 7 International Rescue Committee, New York, New York, United States of America; International Organization for Migration, SRI LANKA

## Abstract

**Background:**

Adolescent girls in humanitarian settings are especially vulnerable as their support systems are often disrupted. More than 20 years of violence in the Democratic Republic of the Congo (DRC) has weakened the health system, resulting in poor sexual and reproductive health (SRH) outcomes for women. Little evidence on adolescent contraceptive use in humanitarian settings is available. CARE, International Rescue Committee (IRC), and Save the Children, in collaboration with the Reproductive Health Access, Information and Services in Emergencies (RAISE) Initiative, Columbia University, have supported the Ministry of Health (MOH) since 2011 to provide good quality contraceptive services in public health facilities in conflict-affected North and South Kivu. In this study, we analyzed contraceptive use among sexually active young women aged 15–24 in the health zones served by the partners’ programs.

**Methods and findings:**

The partners conducted cross-sectional population-based surveys in program areas of North and South Kivu using two-stage cluster sampling in six health zones in July–August 2016 and 2017. Twenty-five clusters were selected in each health zone, 22 households in each cluster, and one woman of reproductive age (15–49 years) was randomly selected in each household. This manuscript presents results from a secondary data analysis for 1,022 women aged 15–24 who reported ever having sex: 326 adolescents (15–19 years) and 696 young women (20–24 years), 31.7% (95% confidence interval [CI] 29.5–34.1), of whom were displaced at least once in the previous five years. Contraceptive knowledge was high, with over 90% of both groups able to name at least one modern contraceptive method. Despite this high knowledge, unmet need for contraception was also high: 31.7% (95%CI 27.9–35.7) among 15–19-year-olds and 40.1% (95% CI 37.1–43.1, *p* = 0.001) among 20–24-year-olds. Current modern contraceptive use (16.5%, 95% CI 14.7–18.4) was similar in both age groups, the majority of whom received their method from a supported health facility. Among current users, more than half of 15–19-year-olds were using a long-acting reversible contraceptive (LARC; 51.7%, 95% CI 41.1–61.9) compared to 36.5% of 20–24-year-olds (95% CI 29.6–43.9, *p* = 0.02). Age, younger age of sexual debut, having some secondary education, being unmarried, and having begun childbearing were associated with modern contraceptive use. The main limitations of our study are related to insecurity in three health zones that prevented access to some villages, reducing the representativeness of our data, and our defining sexually active women as those who have ever had sex.

**Conclusions:**

In this study, to our knowledge one of the first to measure contraceptive prevalence among adolescents in a humanitarian setting, we observed that adolescent and young women will use modern contraception, including long-acting methods. Meaningful engagement of adolescent and young women would likely contribute to even better outcomes. Creating an enabling environment by addressing gender and social norms, however, is key to reducing stigma and meeting the demand for contraception of young women. As we continue to build such supportive environments, we can see that they will use effective contraception when contraceptive services, including short- and long-acting methods, are available, even in protracted crisis settings.

## Abstract 2

In a cross-sectional population-based survey, Sara E. Casey and colleagues assess contraceptive use among adolescent and young women in North and South Kivu, Democratic Republic of the Congo.

## Author summary

**Why was this study done?:**

- Adolescent girls are particularly vulnerable in humanitarian crises as their support systems are often disrupted: they may be separated from their families or communities, educational programs may be interrupted, and social networks may break down.
- Access to contraception is often limited in humanitarian settings, even more so for adolescent and young women.
- Little evidence on adolescent and young women’s contraceptive use in humanitarian settings is available.

**What did the researchers do and find?:**

- We conducted secondary analysis of data on 1,022 sexually active adolescent (15–19 years) and young women (20–24 years) aged 15–24 years from a cross-sectional survey of women of reproductive age in six health zones of conflict-affected North and South Kivu, DRC, where program partners supported the Ministry of Health to provide contraceptive services.
- In these locations, we found that 16.5% of sexually active adolescents and young women were using a modern contraceptive.
- Half of contraceptive users aged 15–19 years were using a long-acting reversible contraceptive.
- Contraceptive knowledge was high (over 90%), but so was unmet need for contraception (over 30%).

**What do these findings mean?:**

- Young women will use modern contraceptive services when they are available.
- A full range of contraceptive methods, including long-acting methods, should be made available to women and girls in humanitarian settings.
- These results provide informative considerations for future adolescent sexual and reproductive health programming in fragile contexts and show the need to build supportive environments for adolescents and young people to access contraception in an informed, unbiased manner.
- The main limitations of the study include insecurity in three of six health zones that prevented access to some villages, reducing the representativeness of our data, and our defining sexually active women as those who have ever had sex due to reluctance among women to report date of last sexual activity.

## Introduction

Adolescents comprise a large proportion of the population globally—making up 23% of the population in the least developed countries, where the majority of humanitarian emergencies occur [1]. In addition, adolescents’ sexual and reproductive health (SRH) needs remain largely unmet globally. As of 2017, an estimated 36 million adolescent girls aged 15–19 years old were married or sexually active and did not want to become pregnant in the next two years while more than half of them, approximately 20 million girls, were not using, but were in need of, a modern contraceptive method [2]. Adolescence is a unique period of transition between childhood and adulthood, an important time to shape behavior and norms, including the development of positive practices for future good health and well-being [3]. Adolescents in humanitarian settings may be significantly impacted as their support systems are often disrupted by crisis, exposing them to sexual coercion, exploitation and violence, early marriage, and other negative coping mechanisms [4]. These can result in increases in unintended pregnancies and demand for safe abortion care, among other outcomes [5]. While *the Inter-Agency Field Manual for Reproductive Health in Humanitarian Settings* provides helpful guidance on adolescent-inclusive programming in line with the Minimum Initial Service Package for SRH in crisis, adolescent-friendly SRH services are limited in humanitarian settings [6]. The evidence base on best practices and lessons learned for adolescent SRH largely comes from development contexts; few data on adolescent contraceptive use or preferences are available in humanitarian settings, where access to contraception, and especially long-acting contraceptives, is often limited for all women [6–9]. The 2018 revision of the *Inter-Agency Field Manual* acknowledges the importance of contraception in reducing maternal mortality in humanitarian crises [5].

Given this situation, it is critical to disseminate and build upon learning on adolescent SRH in fragile contexts such as the Democratic Republic of the Congo (DRC). More than 20 years of political instability and insecurity have severely weakened the health system in the DRC, especially in North and South Kivu, which have experienced regular cycles of instability and violence, including significant population movement following the 1994 Rwandan genocide. The impact of the crisis on women can be seen in the poor health outcomes such as DRC’s high maternal mortality ratio of 846 maternal deaths per 100,000 live births and low modern contraceptive prevalence of 7.5% [10]. According to the 2014 Demographic and Health Survey (DHS), 18.0% and 20.7% of adolescents aged 15–19 had begun childbearing in North and South Kivu, respectively [10]. Stigma related to pregnancy outside of marriage or being a “girl-mother” is high in North and South Kivu [11,12]. In Masisi, North Kivu, a 2013 assessment found that none of the 26 assessed public health facilities had a health worker trained to provide adolescent-friendly services; participants in focus group discussions stated that adolescent women were less likely than adult women to access contraceptive services [7]. This is consistent with other studies that also show that adolescent-friendly SRH services were limited nationwide [13,14].

Given the substantial SRH needs in crisis-affected North and South Kivu, CARE, International Rescue Committee (IRC), and Save the Children, in collaboration with the Reproductive Health Access, Information and Services in Emergencies (RAISE) Initiative at Columbia University, began supporting the Ministry of Health (MOH) to provide SRH services in 2007. Since 2011, the partners focused on provision of good quality contraceptive and postabortion care services in public health facilities. As described elsewhere, support to the MOH included competency-based clinical training and supportive supervision, assurance of supplies and infrastructure, community collaboration and mobilization, and consistent data management for ongoing monitoring, evaluation, and data use, with a strong emphasis on improving quality of care [15–19]. When quality of care challenges were identified, the partners addressed them by, for example, improving training on counseling, improved coaching of providers to improve competence and values clarification, and attitudes transformation activities [20]. All contraceptive services were provided free of charge. Program reviews in 2015 found contraceptive services to be good quality and consistently available in the supported health facilities [21]. Although few adolescent-specific activities were implemented in the early years of the programs, the partners worked to improve provider attitudes and reduce bias related to unmarried and adolescent women’s access to SRH services. In 2016 and 2017, the partners conducted a program evaluation to measure contraceptive prevalence in program areas of North and South Kivu. Results from the full sample have been presented elsewhere [17]; this secondary analysis of the evaluation data presents contraceptive prevalence among adolescent and young women aged 15–24 years.

## Methods

### Study design and sample

The partners conducted cross-sectional population-based surveys in program areas of six rural health zones of North Kivu (Kayna, Lubero, Masisi, and Mweso) and South Kivu (Kabare and Kalehe). Two-stage cluster sampling was used to ensure representation of program areas in each health zone. Using probability proportional to size, 25 clusters were selected in each health zone (26 in two highly insecure zones). In each cluster, 22 households were systematically selected; one woman aged 15–49 years was randomly selected in each household. Data were weighted according to the number of eligible women of reproductive age in each household. Detailed methodology for this survey was previously described in another manuscript [17]. This manuscript, which does not have a prospective protocol, details a secondary data analysis from a subgroup of the total sample of 3,271 women of reproductive age: 1,022 adolescent and young women aged 15–24 years old who reported sexual activity, defined as having ever had sex.

### Study procedures

The study questionnaire was adapted from instruments of the *Demographic and Health Surveys*, Ipas, and RAISE and covered women’s knowledge, attitudes, and behaviors related to contraception and abortion. The questionnaire (S1 Text) was developed in French and translated into Congolese Swahili and Kinyarwanda; the translation was reviewed and revised by the survey teams. The questionnaire was piloted in villages not included in the survey sample.

Paper questionnaires were used in four health zones; tablets using KoboToolbox (Harvard Humanitarian Initiative, Cambridge, MA) were used in two health zones. Female interviewers were recruited locally and trained on the survey questionnaire and respondent selection. During training, interviewers discussed the different terms used in French and local languages for contraceptive methods to ensure accurate identification of the method used. Supervisors reviewed paper questionnaires in the field for accuracy and asked the interviewer to return if clarification was needed. Tablet data were uploaded each evening for review by the first author and discussed with the supervisors each morning. Data collection took place in July–August 2016 in four health zones and July–August 2017 in two health zones.

### Ethical considerations

All respondents provided oral informed consent; names were not entered onto questionnaires or consent forms to preserve anonymity. The study received a waiver of parental consent for 15–17-year-olds as it met the criteria for minimal risk. Ethical approvals for the survey were provided by the Institutional Review Board of the Mailman School of Public Health, Columbia University (AAAQ7431), and the Institutional Ethical Commission of the Catholic University of Bukavu in DRC (UCB/CIE/NC/009/2016).

### Analysis

Data were entered into CSPro 6.0 or KoboToolbox (KoBoCollect 1.4.8) and subsequently exported to PASW (SPSS) Version 24 for cleaning and analysis. Sociodemographic characteristics were stratified by age group and reported with 95% confidence intervals (CIs). Chi-squared (categorical variables) and *t* tests (means) were used to describe and compare results between age groups; these are reported with 95% CI and *p*-values. Observations with missing data for specific variables were excluded from analysis of those variables; missing data are ≤1% for all variables except duration of current displacement and timing of last sexual activity (1.2% of weighted base). The primary outcome measures were current use of modern contraceptives and current use of a long-acting reversible contraceptive (LARC). Modern contraceptive methods were defined as tubal ligation, vasectomy, intrauterine devices (IUDs), implants, injectables, oral contraceptive pills, and male and female condoms; LARCs were defined as IUD and implant. Contraceptive knowledge included both spontaneous and prompted knowledge of individual contraceptive methods. Unmet need for contraception was defined as married or sexually active fecund women who are not currently using contraception and who do not want to be pregnant within two years or ever. Statements regarding attitudes or satisfaction were asked on a four-point Likert scale and collapsed into two categories (agree/disagree and satisfied/not satisfied).

Logistic regression was used to calculate odds ratios (ORs) and 95% CI to determine factors associated with modern contraceptive use in this population. Independent variables in the model included proximate determinants of fertility such as age (continuous variable), religion (Catholic, Protestant, other/none), marital status (not married, currently married, or cohabitating), education (no formal schooling, some or completed primary. some or completed secondary or higher), displaced at least once in the previous 5 years (yes/no), age at first sexual encounter (continuous variable), and had begun childbearing (yes/no). This study is reported as per the Strengthening the Reporting of Observational Studies in Epidemiology (STROBE) guidelines (S1 STROBE Checklist).

## Results

The total sample size was 1,022 sexually active women with 326 adolescents (15–19 years) and 696 young women (20–24-year-olds). Sociodemographic characteristics were similar across both age groups: 44.9% (95% CI 42.4.1–47.3) said they were Catholic and 41.5% Protestant (95% CI 39.1–44.0) (Table 1). One third (31.7%, 95% CI 29.5–34.1) reported being displaced at least once in the previous five years, and 16.1% (95% CI 14.3–18.0) reported being displaced at the time of the survey. Less than half of the sample (43.0%, 95% CI 40.6–45.5) reported having some or completed secondary school, while 23.2% (95% CI 21.1–25.4) reported no formal schooling. More 15–19-year-olds (20.2%, 95% CI 16.7–24.3) than 20–24-year-olds (11.2%, 95% CI 14.4–19.0) reported being currently in school.

**Table 1:** Sociodemographic characteristics^1^.

Characteristic	Total (*N* = 1,561, 1,022)^2^ [95% CI] (*n*)	*15–19-year-olds* (*N* = 543, 326)^2^ [95% CI] (*n*)	*20–24-year-olds* (*N* = 1,018, 696)^2^ [95% CI] (*n*)
**Age at first sexual intercourse**			
Mean age (standard deviation, SD), range, years	16.4 (2.2), 8–24	15.4 (1.9), 8–19	16.9 (2.2), 9–24
Under 15 years	16.2% [14.4–18.1] (143)	26.4% [22.9–30.3] (75)	10.7% [8.9–12.8] (68)
15–17 years	52.7% [50.2–55.1] (532)	61.1% [56.9–65.1] (207)	48.2% [45.1–51.3] (325)
18–24 years	31.2% [28.9–33.5] (339)	12.5% [10.0–15.5] (42)	41.1% [38.1–44.2] (297)
**Age at first marriage**			
Mean age (SD), range, years	17.3 (2.1), 10–24	16.2 (1.6), 10–19	17.8 (2.2), 12–24
Under 15	6.6% [5.3–8.3] (44)	12.6% [9.3–16.8] (21)	4.3% [3.1–6.0] (23)
15–17 years	45.5% [42.6–48.6] (359)	66.2% [60.7–71.3] (142)	37.4% [34.1–40.9] (217)
18–24 years	47.9% [44.8–50.8] (367)	21.2% [17.0–26.2] (42)	58.3% [54.8–61.7] (325)
**Education**			
None	23.2% [21.1–25.4] (268)	20.8% [17.6–24.5] (72)	24.5% [22.0–27.3] (196)
Some or completed primary school	33.7% [31.4–36.1] (352)	36.6% [32.7–40.8] (124)	32.2% [29.4–35.2] (228)
Some or completed secondary school or higher	43.0% [40.6–45.5] (398)	42.6% [38.5–46.8] (129)	43.3% [40.2–46.3] (269)
**Currently in school**	14.4% [12.6–16.5] (78)	20.2% [16.7–24.3] (35)	11.2% [9.2–13.6] (43)
**Displaced at least once in previous 5 years**	31.7% [29.5–34.1] (350)	31.2% [27.4–35.2] (112)	32.1% [29.3–35.0] (238)
Displaced now	16.1% [14.3–18.0] (208)	15.2% [12.4–18.5] (80)	16.5% [14.4–19.0] (128)
**Marital status**			
Married or cohabiting	61.2% [58.8–63.6] (716)	47.1% [43.0–51.4] (186)	68.8% [65.9–71.6] (530)
Not married or cohabiting	38.8% [36.4–41.2] (621)	52.9% [48.7–57.0] (404)	31.2% [28.5–34.2] (217)
**Has begun childbearing**	84.8% [83.0–86.5] (910)	75.7% [71.9–79.1] (263)	89.7% [87.7–91.4] (647)
**Currently pregnant**	20.3% [18.4–22.4] (221)	22.3% [19.0–26.0] (83)	19.3% [17.0–21.8] (138)
**Has had an unintended pregnancy in her life**	45.1% [42.7–47.6] (469)	41.1% [37.1–45.3] (136)	47.3% [44.2–50.4] (333)
**Number of lifetime pregnancies**			
None	15.2% [13.5–17.1] (112)	24.3% [20.9–28.1] (63)	10.3% [8.6–12.3] (49)
1–3	72.3% [70.0–74.4] (756)	72.6% [68.7–76.1] (251)	72.1% [69.3–74.8] (505)
4 or more	12.6% [11.0–14.3] (154)	3.1% [2.0–5.0] (12)	17.6% [15.4–20.0] (142)
**Religion**			
Catholic	44.9% [42.4–47.3] (423)	45.2% [41.1–49.4] (130)	44.7% [41.6–47.7] (293)
Protestant	41.5% [39.1–44.0] (448)	38.6% [34.6–42.7] (138)	43.1% [40.1–46.2] (310)
Other or no religion	13.6% [12.0–15.4] (147)	16.2% [13.4–19.6] (57)	12.3% [10.4–14.4] (90)

^1^Values are weighted percentages, [95% CIs] (absolute counts). Chi-squared (categorical variables) and *t* tests (means) were used to compare results between age groups.

^2^*N* = weighted and unweighted base.

Abbreviation: CI, confidence interval

Age of sexual debut was 15.4 years among sexually active 15–19-year-olds and 16.9 years among sexually active 20–24-year-olds; 26.4% (95% CI 22.9–30.3) of 15–19-year-olds and 10.7% (95% CI 8.9–12.8) of 20–24-year-olds said they first had sexual intercourse before age 15. Nearly half of 15–19-year-olds (47.1%, 95% CI 43.0–51.4) and 68.8% (95% CI 65.9–71.6) of 20–24-year-olds reported being currently married or cohabiting with the mean age at marriage of 16.2 (15–19-year-olds) and 17.8 (20–24-year-olds). The majority of our sample had begun childbearing: 75.7% (95% CI 71.9–79.1) of 15–19-year-olds and 89.7% (95% CI 87.7–91.4) of 20–24-year-olds, with 17.6% (95% CI 15.4–20.0) of 20–24-year-olds reporting four or more pregnancies. Nearly half of respondents (41.1%, 95% CI 37.1–45.3, of 15–19-year-olds; 47.3%, 95% CI 44.2–50.4, of 20–24-year-olds) reported a previous unintended pregnancy, 91.9% (95% CI 89.7–93.7) of whom reported at least one living child.

Contraceptive knowledge was high in the sample, with over 95.3% (95% CI 94.1–96.2) able to name at least one modern contraceptive method and 83.8% (95% CI 80.5–86.7) of 15–19-year-olds and 87.5% (95% CI 85.3–89.4) of 20–24-year-olds able to name any LARC or permanent method (Table 2). The older age group was more likely than younger women to say they received contraceptive information from a health facility or health worker (75.7%, 95% CI 72.9–78.2 compared to 62.3%, 95% CI 58.1–66.3) or from a community health worker (CHW; 29.8%, 95% CI 27.1–32.7 versus 22.4%, 95% CI 19.1–26.1). Friends and family were the second most common source of information in both groups (54.1%, 95% CI 51.6–56.5), followed by the radio (30.8%, 95% CI 28.6–33.2). Attitudes towards contraception overall were favorable, with 85.4% (95% CI 83.5–87.0) agreeing that contraception helps a couple take better care of their family, and 76.9% (95% CI 74.7–78.9) disagreeing that contraception may cause fertility problems. Attitudes towards adolescent access were somewhat more mixed. While 69.0% (95% CI 66.6–71.2) of respondents agreed that adolescent women need to know how to prevent pregnancies, just over half (53.4% [95% CI 49.2–57.6] of 15–19-year-olds and 60.2% [95% CI 57.2–63.2] of 20–24-year-olds) agreed that adolescent women should be allowed to obtain contraception if they want.

**Table 2:** Contraceptive knowledge and attitudes^1^.

Knowledge or attitude	Total (*N* = 1,561, 1,022)^2^ [95% CI] (*n*)	*15–19-year-olds* (*N* = 543, 326)^2^ [95% CI] (*n*)	*20–24-year-olds* (*N* = 1,018, 696)^2^ [95% CI] (*n*)	*p*-value
**Knowledge of any modern method**	95.3% [94.1–96.2] (977)	94.3% [92.0–96.0] (307)	95.8% [94.4–96.9] (670)	0.19
**Knowledge of any long-acting or permanent method**	86.2% [84.4–87.8] (895)	83.8% [80.5–86.7] (276)	87.5% [85.3–89.4] (619)	0.04
**Source of contraceptive information**				
Health facility/health worker	71.0% [68.7–73.2] (744)	62.3% [58.1–66.3] (209)	75.7% [72.9–78.2] (535)	<0.001
Friend, family member	54.1% [51.6–56.5] (530)	56.6% [52.4–60.7] (183)	52.7% [49.6–55.8] (347)	0.66
Radio	30.8% [28.6–33.2] (287)	31.8% [28.0–35.8] (91)	30.3% [27.5–33.2] (196)	0.99
CHW	27.2% [25.0–29.5] (279)	22.4% [19.1–26.1] (74)	29.8% [27.1–32.7] (205)	0.005
Other	11.5% [10.0–13.2] (113)	11.8% [9.4–14.8] (39)	11.3% [9.5–13.4] (74)	0.09
***Favorable attitudes towards contraception***				
Contraceptive use helps a couple take better care of their family. (Agreed)	85.4% [83.5–87.0] (885)	84.7% [81.4–87.5] (281)	85.7% [83.5–87.8] (604)	0.58
A woman who uses contraception may have trouble getting pregnant again. (Disagreed)	76.9% [74.7–78.9] (774)	75.0% [71.2–78.5] (240)	77.9% [75.2–80.4] (534)	0.20
Adolescent women need to know how to prevent pregnancies. (Agreed)	69.0% [66.6–71.2] (672)	66.9% [62.8–70.7] (209)	70.1% [67.2–72.8] (463)	0.19
Adolescent women should be allowed to obtain contraception if they want. (Agreed)	57.8% [55.4–60.3] (574)	53.4% [49.2–57.6] (176)	60.2% [57.2–63.2] (398)	0.01

^1^Values are weighted percentages, [95% CIs] (absolute counts); chi-squared tests were used to compare results between age groups.

^2^*N* = weighted and unweighted base.

Abbreviation: CHW, community health worker

Unmet need for contraception in this population was 31.7% (95% CI 27.9–35.7) among 15-19-year-olds and 40.1% (95% CI 37.1–43.1) among 20–24-year-olds (Table 3). Reported ever use of a modern method was 28.9% (95% CI 25.3–32.9) among 15–19-year-olds and 33.9% (95% CI 31.1–36.9) among 20–24-year-olds; ever use of a LARC was similar in both groups at 11.6% (95% CI 10.1–13.3). Current modern contraceptive use (16.5% 95% CI 14.7–18.4) and LARC use (6.9%, 95% CI 5.7–8.2) were similar in both groups. Among those using a modern method, more than half of 15–19-year-olds were using a LARC (51.7%, 95% CI 41.4–61.9) compared to 36.5% (95% CI 29.6–43.9) of 20–24-year-olds (*p* = 0.02). Overall, implants were the most commonly used method followed by condoms, and then IUDs for 15–19-year-olds and injectables for 20–24-year-olds. Most women (64.3%, 95% CI 58.2–70.0) received their method from a supported health facility. A pharmacy or shop (20.2%, 95% CI 15.7–25.6) was the second most common source; all who received their method at a pharmacy, shop, or other source used condoms or pills. Current modern method use was higher among unmarried women (23.0%, 95% CI 19.8–26.5) than among married women (12.3%, 95% CI 10.3–14.5, *p* < 0.001); current use of a LARC was also higher among unmarried (9.4%, 95% CI 7.4–12.0) compared to married women (5.2%, 95% CI 4.0–6.8, *p* = 0.001).

**Table 3:** Modern contraceptive use^1^.

Outcome	Total (*N* = 1,561, 1,022)^2^ [95% CI] (*n*)	*15–19-year-olds* (*N* = 543, 326)^2^ [95% CI] (*n*)	*20–24-year-olds* (*N* = 1,018, 696)^2^ [95% CI] (*n*)	*p*-value
**Unmet need for contraception**	37.2% [34.8–39.6] (408)	31.7% [27.9–35.7] (105)	40.1% [37.1–43.1] (303)	0.001
**Reported ever use of a modern method**	32.2% [29.9–34.5] (311)	28.9% [25.3–32.9] (85)	33.9% [31.1–36.9] (226)	0.05
**Reported ever use of a LARC**	11.6% [10.1–13.3] (118)	9.8% [7.5–12.6] (31)	12.6% [10.7–14.8] (87)	0.10
**Current contraceptive use**				
Modern method	16.5% [14.7–18.4] (152)	16.0% [13.2–19.3] (48)	16.7% [14.5–19.1] (104)	0.73
LARC	6.9% [5.7–8.2] (67)	8.3% [6.3–10.9] (26)	6.1% [4.8–7.7] (41)	0.10
*By method*:				0.07
Tubal ligation	0.8% [0.2–2.8] (1)	0.0% [0–4.2] (0)	1.2% [0.3–4.2] (1)	
IUD	8.2% [5.4–12.2] (11)	13.8% [8.1–22.6] (6)	5.3% [2.8–9.8] (5)	
Implant	32.7% [27.2–38.6] (55)	37.9% [28.5–48.4] (20)	30.0% [23.6–37.3] (35)	
Injectable	15.2% [11.3–20.1] (25)	10.3% [5.5–18.5] (4)	17.6% [12.7–24.1] (21)	
Oral contraceptive pills	11.3% [8.0–15.7] (19)	9.2% [4.7–17.1] (5)	12.4% [8.2–18.1] (14)	
Condom	31.9% [26.5–37.8] (41)	28.7% [20.3–39.0] (13)	33.5% [26.9–40.9] (28)	
**First source of current method**				0.70
Supported health facility	64.3% [58.2–70.0] (103)	67.8% [57.4–76.7] (33)	62.4% [54.8–69.5] (70)	
Nonsupported health facility	11.5% [8.1–16.0] (18)	11.5% [6.4–19.9] (6)	11.5% [7.5–17.3] (12)	
Pharmacy or boutique	20.2% [15.7–25.6] (25)	18.4% [11.7–27.8] (7)	21.2% [15.7–28.1] (18)	
Other	4.0% [2.2–7.2] (5)	2.3% [0.6–8.0] (2)	4.8% [2.5–9.3] (3)	
**Received her preferred method**	95.3% [92.0–97.3] (146)	95.4% [88.8–98.2] (46)	95.3% [91.0–97.6] (100)	0.97
**Reported at least one problem with current method**	23.0% [18.2–28.5] (38)	28.7% [20.3–39.0] (12)	20.0% [14.7–26.7] (26)	0.12
**Satisfied with her method**	91.4% [87.4–94.3] (139)	95.4% [88.8–98.2] (46)	89.4% [83.9–93.2] (93)	0.10
**Plans to continue method use**	84.9% [80.0–88.8] (129)	90.8% [82.9–95.3] (44)	81.8% [75.2–87.0] (85)	0.06
**Planned duration of continued method use**				
Less than 2 years	1.4% [0.5–4.1] (3)	1.3% [0.2–6.9] (1)	1.5% [0.4–5.4] (2)	0.92
2–4 years	44.5% [37.9–51.3] (65)	47.4% [36.7–58.4] (23)	42.7% [34.6–51.3] (42)	
5 years or more	41.1% [34.7–47.9] (46)	38.5% [28.5–49.6] (15)	42.7% [34.6–51.3] (31)	
Unsure	12.9% [9.0–18.1] (11)	12.8% [7.1–22.0] (4)	13.0% [8.3–19.8] (7)	

^1^Values are weighted percentages, [95% CIs] (absolute counts); chi-squared tests were used to compare results between age groups.

^2^*N* = weighted and unweighted base.

Abbreviations: LARC, long-acting reversible contraceptive; IUD, intrauterine device

Nearly all respondents (95.3%, 95% CI 82.0–97.3) received their preferred method, and the majority (77.0%, 95% CI 71.5–81.8) reported no problems with the method. The vast majority reported wanting to continue using their method for more than 2 years: 44.5% (95% CI 37.9–51.3) for 2–4 years and 41.1% (95% CI 34.7–47.9) for 5 or more years. Satisfaction with their method was likewise high (91.4%, 95% CI 87.4–94.3).

In our adjusted model (including age, age at first sexual encounter, education, displacement, marital status, childbearing, and religion), primary education, displacement, and religion were not associated with modern contraceptive use (Table 4). Married young women were less likely to use modern contraception (adjusted odds ratio [AOR] 0.44 [95% CI 0.32–0.60]). Having some or completed secondary education was significantly associated with contraception use (AOR 1.77 [95% CI 1.18–2.67]), although some or completed primary school was not. For each year older a woman was at her first sexual encounter, she was 10% less likely to use a modern contraceptive (AOR 0.89 [95% CI 0.83–0.95]). Women who had already begun childbearing were nearly twice as likely to use modern contraception as those who had not (1.73 [95% CI 1.12–2.66]).

**Table 4:** Factors associated with current use of a modern contraceptive method.

Characteristic	Unadjusted OR (95% CI)	AOR (95% CI)	*p*-value, AOR
**Age**	1.05 (0.99–1.11) *p* = 0.14	1.11 (1.04–1.18)	0.002
**Age at first sexual intercourse**	0.89 (0.84–0.95) *p* < 0.001	0.89 (0.83–0.95)	<0.001
**Education**			
None (reference)	1.00		
Some or completed primary	1.58 (1.05–2.36) *p* = 0.027	1.337 (0.88–2.02)	0.18
Some or completed secondary or higher	1.94 (1.33–2.84) *p* = 0.001	1.77 (1.18–2.67)	0.006
**Displaced in previous 5 years**	1.08 (0.81–1.44) *p* = 0.59	1.30 (0.95–1.78)	0.10
**Married or cohabiting**	0.47 (0.36–0.61) *p* < 0.001	0.44 (0.32–0.60)	<0.001
**Has begun childbearing**	1.16 (0.79–1.71) *p* = 0.45	1.73 (1.12–2.66)	0.01
**Religion**			
Catholic (reference)	1.00		
Protestant	1.06 (0.80–1.41) *p* = 0.67	1.00 (0.73–1.36)	1.0
Other/none	0.69 (0.44–1.09) *p* = 0.12	0.73 (0.46–1.18)	0.20

Abbreviations: AOR, adjusted odds ratio; OR, odds ratio

The most commonly cited reason for not using a modern method among nonusers were reasons related to a perceived low risk of pregnancy or a desire to become pregnant, including those who wanted to become pregnant, were pregnant, or were having infrequent or no sex: 64.2% (95% CI 58.9–69.1) among 15–19-year-olds and 43.5% (95% CI 39.7–47.4) among 20–24-year-olds (Table 5). Method-related reasons, including fear of side effects, were the second most commonly mentioned reason (37.6%, 95% CI 34.6–40.7). Opposition to use by the respondent or others was more often cited by 20–24-year-olds (37.2%, 95% CI 33.5–41.1) than by 15–19-year-olds (20.8%, 95% CI 16.8–25.5). This is likely due to the higher proportion of the older group reporting opposition by their husbands or partners (22.0%, 95% CI 19.0–25.4 versus 11.1%, 95% CI 8.2–15.0, *p* < 0.001); opposition by the respondent herself was lower in both groups (7.0%, 95% CI 5.5–8.7).

**Table 5:** Barriers to contraceptive use reported by young women who are not currently using any contraception and are not currently pregnant^1^.

Barrier	Total (*N* = 1,546, 1,014)^2^ [95% CI] (*n*)	*15–19-year-olds* (*N* = 537, 324)^2^ [95% CI] (*n*)	*20–24-year-olds* (*N* = 1,009, 690)^2^ [95% CI] (*n*)	*p*-value
Perceived low risk of or desire for pregnancy^3^	50.6% [47.5–53.8] (289)	64.2% [58.9–69.1] (112)	43.5% [39.7–47.4] (177)	<0.001
Opposition to use^4^	31.6% [28.7–34.6] (227)	20.8% [16.8–25.5] (48)	37.2% [33.5–41.1] (179)	<0.001
Lack of knowledge^5^	10.7% [8.9–12.8] (67)	11.1% [8.2–15.0] (20)	10.4% [8.3–13.0] (47)	0.73
Method-related reasons^6^	37.6% [34.6–40.7] (248)	36.1% [31.2–41.4] (72)	38.3% [34.6–42.2] (176)	0.51
Lack of access^7^	3.2% [2.3–4.5] (19)	2.1% [1.0–4.3] (4)	3.8% [2.6–5.6] (15)	0.16
Other^8^	4.0% [3.0–5.5] (31)	2.1% [1.0–4.3] (6)	5.0% [3.6–7.0] (25)	0.03

^1^Values are weighted percentages, [95% CIs] (absolute counts); chi-squared tests were used to compare results between age groups.

^2^*N* = weighted and unweighted base.

^3^*Perceived low risk of or desire for pregnancy* includes those who want to become pregnant, are not married or whose husband is absent, are not having sex or infrequent sex, are (or her partner is) unable to get pregnant or having difficulty getting pregnant, had a hysterectomy, are postpartum or breastfeeding.

^4^*Opposition to use* includes those who oppose contraceptive use or do not want to use contraception, whose husband opposes or others oppose contraceptive use, report religious prohibition, heard or believe that contraception is bad for her.

^5^*Lack of knowledge* includes those who know no method, know no source of methods, lack information or do not have enough information about contraception, or say they have never heard of contraception.

^6^*Method-related reasons* includes those who fear side effects, say that the method is inconvenient or difficult to use, report health-related reasons, or say that contraception does not work.

^7^*Lack of access* includes those who say that services are too far, her preferred method is not available, it is too expensive, the services are not confidential, the providers have bad attitudes.

^8^*Other* includes those who want to wait for a particular number of births before using, have not yet discussed with husband, do not need contraception.

## Discussion

This study is one of few to describe contraceptive knowledge and use among adolescent and young women in a humanitarian setting where contraceptive services are available. Our study finding of 16.5% modern contraceptive prevalence among sexually active adolescent and young women was slightly lower than prevalence among women 15–49 years in this population [17]. Displacement was not associated with contraceptive use suggesting that young women among both the displaced and host community populations have access to and are using contraceptive services. In this population, displacement usually occurs within the program areas, rather than to distant locations beyond the reach of the program. This is consistent with evidence suggesting that fertility patterns and contraceptive use are influenced less by displacement status and more by sociodemographic factors and access, as in stable populations [22]. Unmet need in this population was over 30% in both age groups. It is notable that over 80% of the sexually active women in our sample have already begun childbearing, and nearly half reported a previous unintended pregnancy. This suggests a need to address additional barriers beyond service availability.

LARC use by the young women in our sample (6.9%), while still low, is higher than that found in many stable populations (2.5% across 46 countries) [23]. While we cannot draw a causal link with our program, it is important to note that program partners provided strong support to the MOH to improve contraceptive service delivery in these areas so that young women likely had better access to LARCs than youth in many other settings do [15–19]; for example, most received their method from a health facility supported by the program partners. It is interesting that 15–19-year-olds had higher LARC use than 20–24-year-olds: implants and IUDs were two of the three most used methods in both age groups. As over 80% of modern method users wanted to continue their method for two or more years, more young women may be interested in using a LARC. This highlights the importance of ensuring young women’s access to the full range of contraceptive methods, including LARC. Distributing short-acting methods like condoms, pills, and injectables in youth centers or schools is insufficient to respond to young women’s needs. Adolescents should have access to a broad range of methods offered by qualified providers. Although adolescents often seek contraception outside of health facilities, the majority of our sample received their method in a public health facility, consistent with other findings in DRC in which some adolescents express a preference for going to a health facility over a pharmacy for contraception [24]. The use of a health facility is likely related to the high LARC use in this population (as LARCs are rarely available outside a health facility), similar to findings in a review of DHS data from adolescents in 33 countries [25].

A majority also said they received information about contraception from a health facility or health worker. Program partners have worked with health workers, discussing provider attitudes towards adolescent or unmarried women’s sexual activity and contraceptive use, which may have contributed to these results. When health workers are supported to provide nonjudgmental good quality services, including skills strengthening and values clarification and attitudes transformation, they can build trust with young women and serve as key sources of information and methods for them. Investments in quality, combined with free care, can therefore improve equity. For short-acting method users, community-level provision may improve their access and use of contraception, although results from such pilots in DRC have thus far been mixed [26,27]. It is, however, important to note that 20–24-year-olds were more likely to receive contraceptive information from health workers or CHWs than 15–19-year-olds. While this could be related to the fact that more 20–24-year-olds are married and having children and therefore spending more time in health facilities, it also suggests that provider bias may continue with respect to unmarried or younger women and that further attention to attitudes is needed.

Method-related reasons, including fear of side effects and beliefs in myths about contraceptives, were the second most common barrier to contraceptive use mentioned in both age groups. Although most women correctly disagreed that contraception causes infertility, other myths about contraceptives may persist that prevent young women from using them. Nearly 60% of our sample reported friends as sources of contraceptive information, suggesting that misinformation is likely to be shared. While knowledge of contraception in this population was generally high, it is concerning that 10% of our sample reported not knowing about contraception. It is likely this lack of knowledge would be even higher among young women who are not yet sexually active. This suggests a need for accurate and complete information about contraceptive methods and outreach to adolescent and young women about contraception to prevent unintended pregnancy, including information on side effects to combat misinformation. It is particularly important to provide correct information to adolescents before they become sexually active, which requires outreach to very young adolescents (10–14-year-olds), something not often done [28].

While women’s attitudes towards contraception were largely favorable, they were least likely to agree that “Adolescent women should be allowed to obtain contraception if they want.” Perhaps surprisingly, fewer women agreed with that statement than agreed that “Adolescent women need to know how to prevent pregnancies.” Despite the similar contraceptive prevalence in both age groups, the 15–19-year-olds were less likely to agree with both statements than the 20–24-year-olds. These findings are consistent with studies showing that adolescents often endorse social norms that reinforce gender inequalities, including a lack of control over their own SRH or lack of empowerment [29,30]. In addition, substantial stigma related to contraceptive use, including that women who use contraception are promiscuous, may contribute to this attitude [24,31–33]. Research suggests that women’s empowerment may be associated with modern contraceptive use [34,35]. Interventions must, therefore, not only address stigma but also create an enabling environment by addressing gender and social norms to help adolescents to recognize and use their agency to make decisions about their SRH. Humanitarian emergencies and the response to them appear to contribute to or accelerate social norm change [36,37], making this a unique time to strengthen adolescent girls’ empowerment.

Age at first marriage was below 18 years in this population of sexually active young women; age of sexual debut was lower. Stigma concerning unmarried adolescent pregnancy is strong, particularly in North and South Kivu, and may contribute to this low age of marriage [11,12]. Girls who get pregnant outside of marriage may be forced by their parents into marriage with the man who got her pregnant [11]. In other cases, “girl-mothers” may be expelled from their home, be forced to leave school, and/or may seek a (likely unsafe) abortion [11,12]. Further analysis of these data comparing married and unmarried young women is needed to ensure appropriate targeting of different groups and meeting their different needs.

Both modern method and LARC use were lower among married women in our sample. This could be related to the family or social pressure to produce a first child quickly after marriage [38,39]. Therefore, a clear opening exists to discuss contraception with young women during antenatal care to encourage spacing and delay of a subsequent pregnancy. In addition, respondents in both age groups reported higher opposition to contraceptive use by their husband or partner than by themselves. Husbands are generally perceived as the key decision-makers regarding family size and therefore contraceptive use in DRC, and are often perceived as barriers, suggesting a need for better outreach and education to men and boys about the benefits of contraception [32,40–42].

After these surveys were conducted, the three implementing partners began collecting age-disaggregated data in supported health facilities. They continue to address health worker attitudes towards adolescents and implement community dialogue and awareness sessions with adolescent SRH messaging. The partners have used these findings to inform pilots of various intervention packages to systematically support more adolescent responsive programming, addressing the unique barriers faced by adolescents. These efforts involve adolescents as a key part of designing, monitoring, and implementing programming to inform an adolescent-responsive model for SRH in DRC, which could be adapted for use in other humanitarian settings. Piloting these adolescent SRH packages in locations with strong existing contraceptive service delivery programming is key to success and can inform adolescent programming in other humanitarian settings.

This study has several limitations. Due to reluctance among adolescent and young women to report date of last sexual activity, we have included all women who have ever had sex to approximate sexually active women, meaning our data are less directly comparable to DHS data. In addition, stigma may have contributed to underreporting of sexual activity and contraceptive use by some unmarried women. Insecurity in three of six health zones prevented access to some villages, reducing the likelihood our data are representative of the full program areas. The use of paper questionnaires or tablets in different health zones may have resulted in differences in data quality.

This study found that sexually active adolescents and young women chose to use modern contraceptive methods, including LARCs; however, high unmet need for contraception and high reports of unintended pregnancies persisted in both age groups despite high awareness. These findings provide informative considerations for future adolescent SRH programming in fragile contexts. For example, adolescents and young women will use contraceptive services when they are available and good quality; meaningful engagement of adolescent and young women would likely contribute to even better outcomes. Community mobilization among adolescent girls and boys as well as adults, including gender and social norms change, however, are key to meeting the demand for contraception of young women. More gender-transformative approaches, including thoughtful collaborations with other sectors (e.g., education, protection) should be explored. As we continue to build supportive environments for adolescents and young people to access contraception in an informed, unbiased manner, we can see that they will use effective contraception when contraceptive services, including short- and long-acting methods, are available, even in ongoing protracted crisis settings.

## Supporting information

S1 STROBE checklist STROBE, Strengthening the Reporting of Observational Studies in Epidemiology. (DOC)

S1 Text Survey questions. (XLSX)

## Abbreviations

AOR: adjusted odds ratio
CHW: community health worker
CI: confidence interval
DHS: Demographic and Health Survey
DRC: Democratic Republic of the Congo
IRC: International Rescue Committee
IUD: intrauterine device
LARC: long-acting reversible contraceptive
MOH: Ministry of Health
OR: odds ratio
RAISE: Reproductive Health Access, Information and Services in Emergencies
SRH: sexual and reproductive health
STROBE: Strengthening the Reporting of Observational Studies in Epidemiology
