# Supplementary material for: Contraceptive use among adolescent and young women in North and South Kivu, Democratic Republic of the Congo: A cross-sectional population-based survey
Source: PLoS Med. 2020 Mar 31;17(3):e1003086. doi: 10.1371/journal.pmed.1003086 (PMC7108687; doi:10.1371/journal.pmed.1003086)
Supplement: S1 STROBE checklist — (DOC) [file pmed.1003086.s001.doc]

STROBE Statement—Checklist of items that should be included in reports of ***cross-sectional studies***

|  | Item No | Recommendation |
| --- | --- | --- |
| **Title and abstract** | 1 | (*a*) Indicate the study’s design with a commonly used term in the title or the abstract  **- Please see title – “a cross-sectional population-based survey”** |
|  |  | (*b*) Provide in the abstract an informative and balanced summary of what was done and what was found  **- Please see abstract** |
| Introduction | | |
| Background/rationale | 2 | Explain the scientific background and rationale for the investigation being reported  **Introduction – paragraphs 1-2** |
| Objectives | 3 | State specific objectives, including any prespecified hypotheses  **Introduction – paragraph 3** |
| Methods | | |
| Study design | 4 | Present key elements of study design early in the paper  **Methods – paragraph 1** |
| Setting | 5 | Describe the setting, locations, and relevant dates, including periods of recruitment, exposure, follow-up, and data collection  **Methods – paragraphs 1-3** |
| Participants | 6 | (*a*) Give the eligibility criteria, and the sources and methods of selection of participants  **Methods – paragraph 1** |
| Variables | 7 | Clearly define all outcomes, exposures, predictors, potential confounders, and effect modifiers. Give diagnostic criteria, if applicable  **Methods – paragraph 5** |
| Data sources/ measurement | 8* | For each variable of interest, give sources of data and details of methods of assessment (measurement). Describe comparability of assessment methods if there is more than one group  **Methods – paragraphs 5-6** |
| Bias | 9 | Describe any efforts to address potential sources of bias  **Methods – paragraphs 2-3** |
| Study size | 10 | Explain how the study size was arrived at  **Methods – paragraph 1** |
| Quantitative variables | 11 | Explain how quantitative variables were handled in the analyses. If applicable, describe which groupings were chosen and why  **Methods – paragraphs 5-6** |
| Statistical methods | 12 | (*a*) Describe all statistical methods, including those used to control for confounding  **Methods – paragraphs 5-6** |
|  |  | (*b*) Describe any methods used to examine subgroups and interactions  **Methods – paragraphs 5-6** |
|  |  | (*c*) Explain how missing data were addressed  **Methods – paragraphs 5** |
|  |  | (*d*) If applicable, describe analytical methods taking account of sampling strategy  **Methods – paragraph 1** |
|  |  | (*e*) Describe any sensitivity analyses |
| Results | | |
| Participants | 13* | (a) Report numbers of individuals at each stage of study—eg numbers potentially eligible, examined for eligibility, confirmed eligible, included in the study, completing follow-up, and analysed  **Results – paragraph 1** |
|  |  | (b) Give reasons for non-participation at each stage |
|  |  | (c) Consider use of a flow diagram |
| Descriptive data | 14* | (a) Give characteristics of study participants (eg demographic, clinical, social) and information on exposures and potential confounders  **Results – paragraphs 1-2** |
|  |  | (b) Indicate number of participants with missing data for each variable of interest |
| Outcome data | 15* | Report numbers of outcome events or summary measures  **Results – paragraphs 3-7** |
| Main results | 16 | *(*a) Give unadjusted estimates and, if applicable, confounder-adjusted estimates and their precision (eg, 95% confidence interval). Make clear which confounders were adjusted for and why they were included  **Results – paragraphs 3-7** |
|  |  | (*b*) Report category boundaries when continuous variables were categorized  **Results – Table 1** |
|  |  | (*c*) If relevant, consider translating estimates of relative risk into absolute risk for a meaningful time period |
| Other analyses | 17 | Report other analyses done—eg analyses of subgroups and interactions, and sensitivity analyses  **Results – paragraphs 6-7** |
| Discussion | | |
| Key results | 18 | Summarise key results with reference to study objectives  **Discussion – paragraphs 1-6** |
| Limitations | 19 | Discuss limitations of the study, taking into account sources of potential bias or imprecision. Discuss both direction and magnitude of any potential bias  **Discussion – paragraph 8** |
| Interpretation | 20 | Give a cautious overall interpretation of results considering objectives, limitations, multiplicity of analyses, results from similar studies, and other relevant evidence  **Discussion – paragraph 9** |
| Generalisability | 21 | Discuss the generalisability (external validity) of the study results  **Discussion – paragraph 9** |
| Other information | | |
| Funding | 22 | Give the source of funding and the role of the funders for the present study and, if applicable, for the original study on which the present article is based  **Funding statement** |
